# Laparoscopic revision after one anastomosis gastric bypass (OAGB): a 4-years experience in a single high-volume bariatric surgery center in northern Italy

**DOI:** 10.1007/s13304-025-02329-4

**Published:** 2025-08-02

**Authors:** Luigi Eduardo Conte, Bruno Sensi, Giulia Griguolo, Michela Orsi, Francesco Cutrupi, Francesca Serio, Giulia Conti, Michela Campanelli, Domenico Benavoli, Paolo Gentileschi

**Affiliations:** 1 University of Rome “Tor Vergata”, Rome, Italy; 2 Unità di Chirurgia Generale Ospedale San Giovanni Decollato-Andosilla, Civita Castellana, Viterbo Italy; 3 Campus Bio-Medico University, Rome, Italy; 4 Department of Bariatric and Metabolic Surgery, Maria Cecilia Hospital, GVM Care and Research, Cotignola, Ravenna Italy

**Keywords:** Bariatric surgery, Revisional surgery, Surgery, Obesity, OAGB, Bypass

## Abstract

One anastomosis gastric bypass (OAGB) is gaining popularity among bariatric procedures. However, data on the number and outcomes of revisional surgeries is scarce. This study included patients undergoing OAGB revision in a high-volume centre between January 2020 and October 2024. The study evaluates the indication for revision, the type of procedure performed, and the success of revisional surgery, assessed by symptom resolution or percent excess weight loss (%EWL) > 40% at 2 years. OAGB was performed on 3280 patients, of which 52 (1.6%) necessitated surgical revision for late complications as well as 18 patients who had their primary OAGB elsewhere. A total of 68 patients (47 females, 21 males) underwent OAGB revision. The mean time to revision after primary OAGB was 28 months. Indications for revision in the 68 patients were recurrent weight gain (51.5%, 0.73% of the total), severe bile reflux (29.4%, 0.52% of the total), marginal ulcers (7.4%, 0.12% of the total), excessive malabsorption (5.9%, 0.06% of the total), and stenosis (5.9%, 0.12% of the total). Revisional procedures in the 68 patients included biliary-pancreatic limb lengthening (47%), conversion to RYGB (29.4%), redo gastro-jejunal anastomosis (13.3%), biliary-pancreatic limb shortening (5.9%), and pouch resizing (4.4%). There were no major postoperative complications and 5.8% minor complications, all managed conservatively. At 20 months, 98.5% of revisional surgeries were successful, with complete symptom resolution for reflux, anastomotic ulcers and stenosis. The mean of %EWL in the recurrent weight gain group was 33.7%, 57.4% and 84% at 3 months, 1 year and 2 years. OAGB appears to be a safe procedure with a low revision rate. When necessary, surgical revision procedures can be tailored and have a high success rates and low morbidity. A management algorithm has been developed and proposed.

## Introduction

One anastomosis gastric bypass (OAGB) was first described by Rutledge et al. in 1997 [1]. This surgical technique entails creating a gastric pouch with a volume of 150–200 ml and a length of no less than 15 cm, where an intestinal loop is anastomosed 150–200 cm distal to Treitz ligament. Compared to Roux-en-Y gastric bypass (RYGB), OAGB is technically simpler, requiring a single anastomosis, leading to reduce operating times. In the literature, the reported revision rate for OAGB appears to range from 2 to 5% but the data is scant [2–6]. The main causes, leading to revision or restoration after OAGB, include inadequate weight loss, or recurrent weight gain (RWG), severe bile reflux, persistent gastroesophageal reflux disease (GERD), persistent marginal ulcer (MU), and protein-calorie malnutrition (PCM) or excessive weight loss. The anatomy of OAGB allows for various options in case of revision, such as conversion to RYGB, elongation of the loop (biliary-pancreatic limb lengthening, BLL), proximalization of the loop (biliary-pancreatic limb shortening, BLS), REDO of the gastro-jejunal anastomosis (RGJA), re-sizing of the gastric pouch (RGP) and restoration to normal anatomy of the alimentary tract (restoration). However, there is little data on outcomes of each of these techniques and this study aims to report the experience of a large volume center and to develop a management algorithm based on its results.

## Methods

This study included patients who underwent revisional bariatric surgery after index OAGB, at the Bariatric and Metabolic Surgery Unit of the Maria Cecilia Hospital in Cotignola (RA), Italy. Patients were identified retrospectively through a prospectively maintained database. The study adhered to the Strengthening the Reporting of Observational Studies in Epidemiology (STROBE) Statement.

### Patient selection

All consecutive patients with available follow-up operated between January 2020 and October 2024 were evaluated for inclusion. Patients older than 18 years of age, who underwent revisional surgery after index OAGB, for any cause, were eligible for inclusion. Any form of revisional BS procedures was accepted as were patients in whom OAGB was already a revisional procedure. Patients with re-operation for early postoperative complications (< 3 months) after index OAGB were excluded as well as those lost to follow-up or who failed to undergo revisional bariatric surgery.

### Study design

Retrospective single center study reporting the short-term outcomes of revisional BS after index OAGB for late complications (> 3 months). Patients with significant complains after OAGB were evaluated for interventions to induce improvement including revisional surgery. Thus, preoperative investigations included: complete blood tests including thorough nutritional evaluation, oral contrast study, endoscopy and, if considered necessary, CT.

Choice of surgical operation was taken by an expert bariatric surgeon based on patient’s history, reason for revision, eating behaviors, pre-operative studies, and personal preference. RWG was defined as gaining > 30% of the initial weight loss [7]. Several different procedures were undertaken for revision of OAGB according to the indication. In particular, BLL was the procedure of choice for RWG or insufficient weight loss, while pouch re-sizing was considered when investigations highlighted a grossly enlarged gastric pouch. RGJA was conceived for patients with anastomotic stenosis or marginal ulcer. Conversion to RYGB was standard in patients with severe gastroesophageal reflux, biliary reflux and was also considered for marginal ulcer. BLS was performed for severe malabsorption syndrome.

### Surgical technique

All surgeries were performed laparoscopically with a 4 trocars technique, with open technique umbilical access.

OAGB was standardized as follows: 10–12 cm long gastric pouch fashioned around a 38 Fr bougie; 200 cm Bilio-Pancreatic Limb (BL); 3–4 cm wide gastro-jejunostomy. Small intestine was brought to the stomach with an ante-colic technique. Anastomosis were performed with a linear stapler and enterotomies closed with barbed running sutures. For patients with BMI > 45 kg/m^2^ or preoperative sporadic symptoms of gastroesophageal reflux, an anti-reflux mechanism was made by a 5–10 cm latero-lateral continuous suture between the afferent BL and gastric pouch. Drainage was used only in cases of complications during the procedure.

Patients with an OAGB performed at other hospitals often exhibit considerable heterogeneity in the length of the BL and the techniques employed for alimentary reconstruction.

Biliary-pancreatic limb lengthening (BLL): The distal end of the gastric pouch is stapled and a new anastomosis performed 50–100 cm more distal, elongating the biliary limb to 250–300 cm.

Biliary-pancreatic limb shortening (BLS): The distal end of the gastric pouch is stapled and a new anastomosis performed 50–100 cm more proximal, shortening the biliary limb to 150–100 cm.

Re-sizing of the gastric pouch (RGP): Refashioning the gastric pouch on its left border, on a 38 Fr Bougie.

REDO of the gastro-jejunal anastomosis (RGJA): Resection of the previous anastomosis using a linear stapler, followed by the creation of a new anastomosis on healthy tissue nearby.

Conversion to RYGB (cRYGB): the biliary limb is separated from the gastro-jejunal anastomosis using a linear stapler. A jejunoileal anastomosis is then created with a linear stapler and barbed sutures, 80 cm from the beginning of the alimentary limb.

**Fig. 1:**
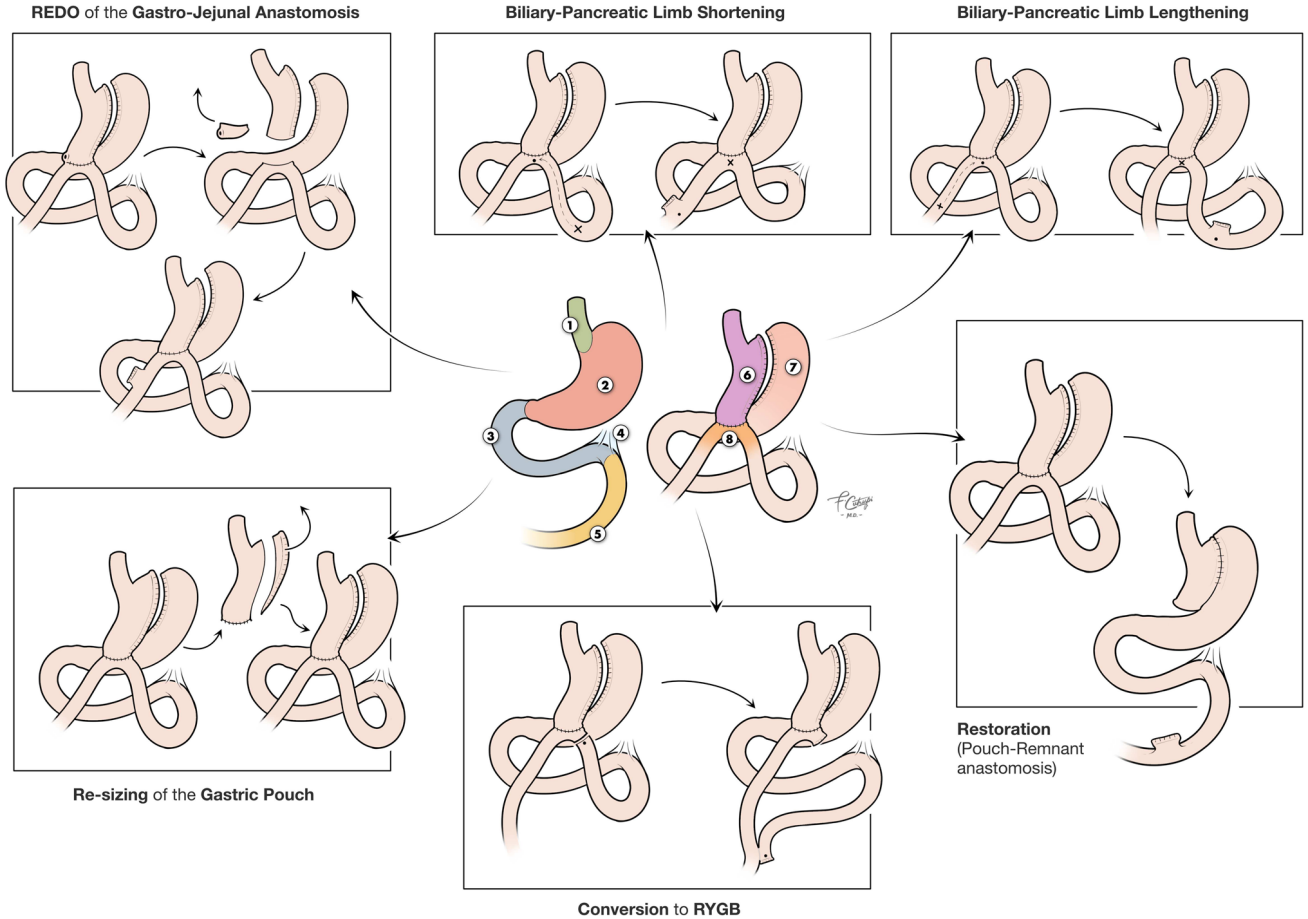
Types of revisions after OAGB: 1—esophagus, 2—stomach, 3—duodenum, 4—Treitz’s ligament, 5—jejunum, 6—gastric pouch, 7—gastric remnant and 8—gastrojejunal anastomosis

Figure 1 summarizes the surgical techniques used.

### Post-operative protocol and follow-up

Re-alimentation protocol was standard with fasting on postoperative day 0, clear liquids on day 1 and 2, semi-liquid diet from day 3 to day 16 and gradual re-introduction of solid foods thereafter. Vitamin supplementation was universal. Follow up included physical examination, surgical, gastroenterological and nutritional evaluation at 1 month, 3 months, 6 months and 12 months from surgical intervention. Some outpatient visits are conducted online to maintain consistent patient follow-up and prevent discontinuity in care and loss to follow-up. Laboratory examination included complete blood count, liver and kidney function, protein electrophoresis and serum vitamin concentrations [8].

### Outcome measures

The primary outcome was incidence of major postoperative complications (Clavien–Dindo > 3a). Secondary outcomes included revisional success, intraoperative complications and BMI at 3 months, 1 year and 2 years of patients with RWG as indication. The percentage of excess weight loss (%EWL) was assessed at 3 months, 1 year, and 2 years after surgery. In patients undergoing conversion for RWG, success was defined as a %EWL > 40% at 2 years [9]. In cases of reflux, stenosis, or marginal ulcer, success was defined as symptom resolution, while in patients with malabsorption, the goal of the revision was nutritional reconstitution.

### Study variables

Pre-operative data included sex, age, BMI, relevant comorbidities (hypertension, DMII, hypercholesterolemia, hypertriglyceridemia, liver steatosis and OSAS), indication for OAGB revision, time to OAGB revision. Intraoperative variables were type of revision and major intraoperative complications (ClassIntra).

Postoperative data included complications expressed as Clavien–Dindo and Comprehensive Complication Index, length of stay and follow-up data (BMI, comorbidities, nutritional status). Liver steatosis was reassessed by abdominal ultrasound at 6 months.

%EWL was calculated as: [(Initial BMI − Final BMI)/(Initial BMI − Ideal BMI] × 100.

Ideal BMI was considered as 25 kg/m^2^.

### Statistical analysis

All quantitative data were expressed as mean ± standard deviation (SD) after testing for normality, while categorical data with percentage frequencies. Categorical data were analyzed using the Chi-square test. Analyses were conducted using SAS, version 9.4 (SAS Institute Inc., Cary, NC, USA) and R, version 4.2 (The R Foundation for Statistical Computing).

### Ethics

This study was conducted according to the international ethical recommendations on clinical research established by the Helsinki and Istanbul Declarations. According to local institutional review board, ethical approval for retrospective studies is not required. All patients signed informed consent for the procedures and for data collection and analyses.

## Results

A total of 3280 patients underwent OAGB at our institution during the study period, of which 52 (1.6%) necessitated surgical revision for late complications. Two patients were excluded because revisional surgery was attempted but aborted due to intraoperative safety concerns. Another 18 patients who underwent OAGB elsewhere underwent OAGB revision at our institution during the study period. Sixty-eight patients had available data and were included in the analysis.

### Patients’s demographics

Baseline characteristics are summarized in Table 1.

**Table 1:** Patients demographics

Patients characteristics	
Median age (± SD)	48.8 (± 8.7) years
Sex (%)	F 47 (69.1%)—M 21 (30.9%)
Mean BMI at revision (± SD)	35 kg/m^2^ (± 8
Mean BMI in WR group	44 kg/m^2^ (± 7 kg/m^2^)
Mean time to revision	28 months
Pre-revision comorbidities	
Arterial hypertension	14 (20.6%)
DM2	4 with oral therapy (5.9%) 1 with insulin therapy (1.5%)
Hepatic steatosis	4 (5.9)
OSAS	1 (1.5%)
Other bariatric procedures before OAGB	
Gastric banding	14 (20.6%)
Sleeve gastrectomy	5 (7.4%)
Gastric banding + sleeve gastrectomy	4 (5.9%)

Median age was 48.8 years (± 8.7 years) and 69.1% of patients were females. Indication for revision was RWG in 35 patients (24 patients operated by our team, 0.73% of the total; 11 operated elsewhere), severe bile reflux in 20 patients (17 patients operated by our team, 0.52% of the total; 3 operated elsewhere), marginal ulcer in five patients (four patients operated by our team, 0.12% of the total; 1 operated elsewhere), excessive malabsorption in four patients (two patients operated by our team, 0.06% of the total; two operated elsewhere), and stenosis in four patients (4 patients operated by our team, 0.12% of the total; 0 operated elsewhere). Mean BMI at revision was 35 kg/m^2^ (± 8 kg/m^2^) for the entire study population and 44 kg/m^2^ (± 7 kg/m^2^) in the RWG group. The mean time to revision after primary OAGB was 28 months. Pre-revision comorbidities in the revised cohort of 68 patients included hepatic steatosis in four patients (5.9%), type 2 diabetes mellitus in four patients on oral hypoglycemic therapy (5.9%) and 1 patient on insulin therapy (1.5%), arterial hypertension in 14 patients (20.6%), and obstructive sleep apnea syndrome (OSAS) in 1 patient (1.5%). Twenty-three patients (33.8%) had underwent other bariatric procedures before OAGB, including 14 gastric banding, 5 sleeve gastrectomy, and four cases of gastric banding followed by sleeve gastrectomy. In this sub-group, RWG was more often the indication for revision compared to other patients (70% vs 45%; *p* = 0.0006).

### Surgical procedures and intraoperative complications

All procedures were performed using a laparoscopic approach. The revisional procedures included BLL in 32 patients (47%), cRYGB in 20 patients (29.4%), RGJA in 9 patients (13.3%), BLS in 4 patients (5.9%), and RGP in 3 patients (4.4%). The 35 patients with RWG as an indication underwent BLL and RGP, while the 20 patients with severe bile reflux underwent cRYGB. The 4 patients with excessive malabsorption underwent BLS, whereas the 9 patients diagnosed with marginal ulcers or stenosis underwent RGJA. Table 2 summarizes the intraoperative procedures performed. There were no major intraoperative complications recorded.

**Table 2:** Surgical procedures and intraoperative complications

Surgical procedure	Indication (*n*)	Number of revised patients (%)	Major intraoperative complications
Biliary-pancreatic limb lengthening	Recurrent weight gain/insufficient weight loss (32)	32 (22 patients operated by our team, 0.67% of the total; 10 operated elsewhere)	0 (0%)
Conversion to RYGB	Severe bile reflux (20)	20 (17 patients operated by our team, 0.52% of the total; 3 operated elsewhere)	0 (0%)
Re-do gastro-jejunal anastomosis	Maginal ulcer—MU (5) Anastomotic stenosis—AS (4)	MU—5 (4 patients operated by our team, 0.12% of the total; 1 operated elsewhere) AS—4 (4 patients operated by our team, 0.12% of the total; 0 operated elsewhere)	0 (0%)
Biliary-pancreatic limb shortening	Malabsorption (4)	4 (2 patients operated by our team, 0.06% of the total; 2 operated elsewhere)	0 (0%)
Resizing gastric pouch	Recurrent weight gain/insufficient weight loss (3)	3 (2 patients operated by our team, 0.06% of the total; 1 operated elsewhere)	0 (0%)

### Postoperative outcomes

Postoperative outcomes are summarized in Table 3.

**Table 3:** Postoperative outcomes

Postoperative outcomes	Number of revised patients
Early complications (< 3 months)	
Minor bleeding	2 (2.9%)
Melena	1 (1.5%)
Fistula	1 (1.5%)
Late complications (> 3 months)	
Bile reflux	3 (4.4%)
Incisional hernia	2 (2.9%)
Protein-calorie malnutrition	1 (1.5%)
POD (± SD)	3.6 (± 4.6)
Additional surgery required	1 restoration for gastric pouch stenosis
BMI at follow-up	
Mean BMI at 3 months	28.8 kg/m^2^ (± 4.8 kg/m^2^)
Mean BMI at 6 months	27.1 kg/m^2^ (± 4.2 kg/m^2^)
Mean BMI at 2 years	27.8 kg/m^2^ (± 5.1 kg/m^2^)
WR group BMI	
Mean BMI at 3 months	37.6 kg/m^2^ (± 7.1 kg/m^2^)
Mean BMI at 6 months	33.1 kg/m^2^ (± 5 kg/m^2^)
Mean BMI at 2 years	28.5 kg/m^2^ (± 8.1 kg/m^2^)
%EWL in WR group	
%EWL at 3 months	33.7%
%EWL at 1 year	57.4%
%EWL at 2 years	84%
Comorbidity outcomes (at 6 months)	
Arterial hypertension	5/14 (35.8%)
Oral hypoglycemic	0/4 (0%)
Insulin therapy	1/1 (100%)
Hepatic steatosis	0/1 (0%)
OSAS	0/1 (0%)

#### Complications and length of stay

There were no major postoperative complications. Minor complications included postoperative anemia (2.9%), melena (1.5%) and postoperative fistula (1.5%) all of which were treated conservatively. There were no other early complications. Late complications included reflux in 4.4% (successfully treated with medications), incisional hernia in 2.9% and one case of pouch stenosis with consequent protein-calorie malnutrition after RGP, which was treated with restorative surgery (i.e., pouch-remnant anastomosis).

Median length of stay was 3.6 ± 4.6 days.

#### Success of revisional BS

All 68 patients correctly attended follow-up. After a mean follow-up of 20 months, revisional BS was successful in 98.5% (*n* 67) of cases. The only patient considered unsuccessful is the one who developed severe malabsorption (RWG/IWL group treated with RGP). In the subgroup of patients selected for RWG, the mean drop in BMI was significant. The mean of the %EWL in the RWG/IWL group was 33.7% at 6 months, 57.4% at 1 year and 84% at 2 years. All cases of reflux were cured with cRYGB. Anastomotic stenosis and marginal ulcers was profitably dealt with RGJA.

None of the patients reported OSAS (0%) or altered fasting blood sugar levels (0%); those previously on oral hypoglycemics had discontinued therapy. The only patient on insulin therapy continued his treatment. Only 5 patients (35.8%) with hypertension showed no improvement in their condition. Liver steatosis was reassessed via abdominal ultrasound at 6 months, with significant improvement in all patients.

## Discussion

One anastomosis gastric bypass is a versatile bariatric surgery procedure that has known large diffusion in recent years and its utilization is ever more frequent. This trend has been confirmed by a number of national and international surveys dealing with surgical demographics [10]. In Italy, for example, OAGB has grown exponentially and represents more than 15% of procedures in the country [11, 12]. However, very long-term data are scarce and there is a paucity of studies on the approach to management of late complications or dissatisfying results. Revisional bariatric surgery is now commonly performed, mainly to deal with sub-optimal outcomes of restrictive procedures such as gastric banding and sleeve gastrectomy [13, 14]. While experience in this regard is now considerable [15], a recent report showed an alarming rate of 10% morbidity and 0.3% mortality [16]. This testifies that today, when choosing a bariatric procedure for a patient, the approach must absolutely be tailored and take into consideration also the long-term trajectories, including the possibility of revisional surgery. Thus, data on revision rates and revision outcomes are crucial. The current study analyzed the experience of a single high-volume centre on OAGB, delivering some very important data.

First, the rate of surgical revision was very low, well below two percent, indicating a reliable procedure which only rarely leads to disappointing outcomes, in line with data from a recent large multi-institutional survey. This is, in the author’s opinion, of great value, especially when compared with outcomes of other more popular procedures such as sleeve gastrectomy [13, 17]. In fact, SG necessitates revision in as many as 10% of patients (with RWG rates up to 70%!) [13, 18]. OAGB, when performed in a standardized fashion in a large volume centre, seems to be highly effective.

**Fig. 2:**
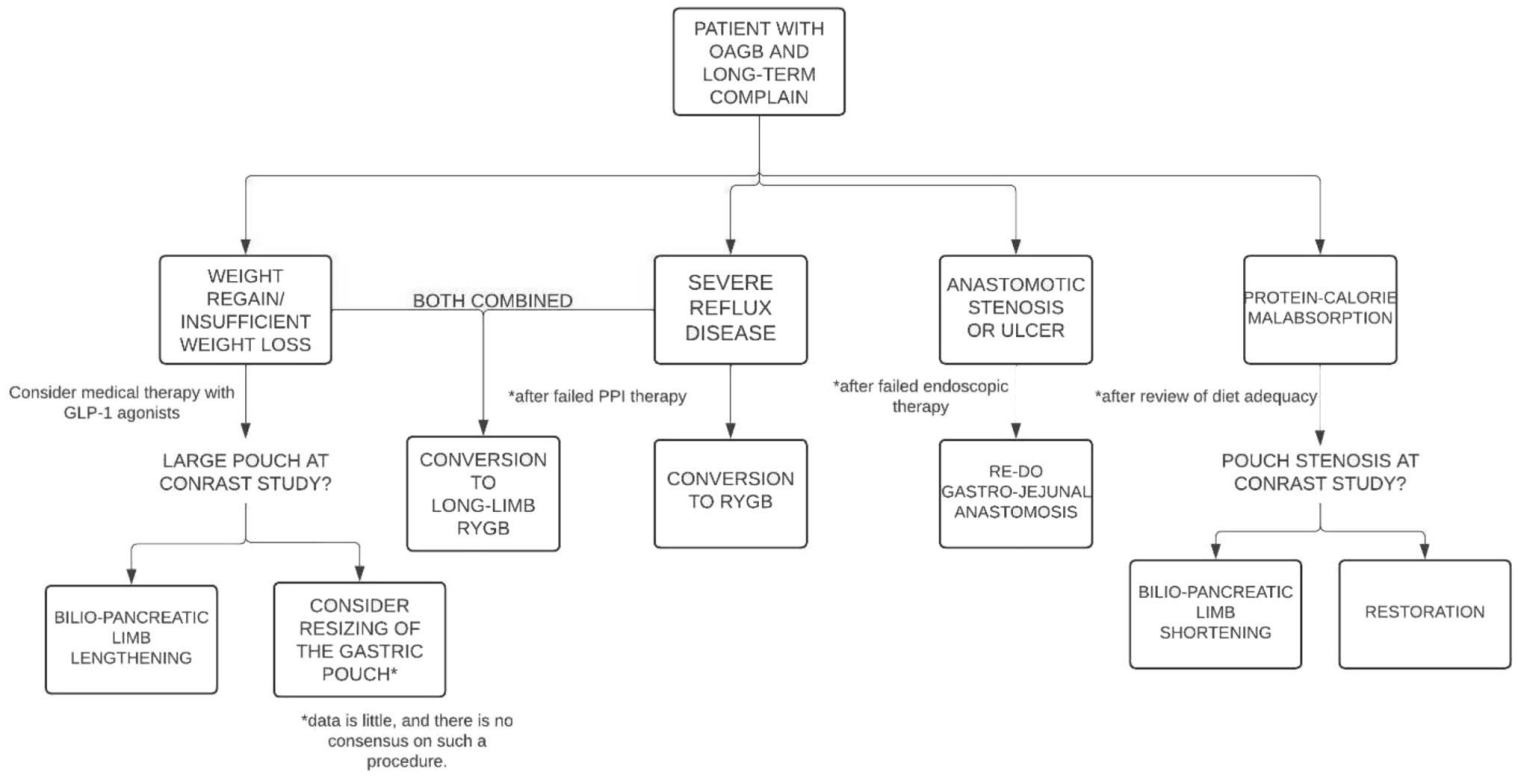
Decision flowchart for OAGB revision type

Second, revisional surgery can be highly successful despite decisional and technical complexity. Here, an approach to the OAGB patient with a long-term complaint, has been developed and proposed based on surgical indication and radiologic anatomy (Fig. 2). RWG/IWL represented the indication for revisional surgery in 51.5% of the cohort. For RWG/IWL, BLL was chosen as the procedure of choice in this study and has performed optimally, with all patients achieving desired weight loss (median 84% EBMIL at 2 years) and the large majority obtaining control on related comorbidities. In fact, this is backed by a recent expert consensus on the matter, which agreed in declaring that BLL is the correct option in this setting [19–21]. The consensus also agreed upon the need to count the whole intestine before BLL, to make sure that sufficient common limb remains to guarantee acceptable absorption. However, the exact length needed is unknown and the consensus advised on maintaning at least 300 to 400 cm of small intestine. In this study, total bowel length was not counted, as it is not during primary operation. The incidence of malabsorption-related syndromes is actually very small (0.06%) and probably quite unpredictable [22]. In fact, when OAGB is standardized and limb lengths are similar to those used for an efficacious RYGB, nutritional status does not seem to be different between OAGB and RYGB [23, 24]. It is conceivable that rigorous follow-up and adequate supplementation may avoid most cases. One patient in this cohort developed protein calorie malnutrition that was related to pouch stenosis and completely unrelated to small bowel length (note that this patient, with the same length of bypassed intestine, had suffered IWL). In fact, in this case the team has chosen to undergo restorative surgery rather than BLS, which was discarded as likely ineffective. This exemplifies underlying complexity and the importance of a large experience, as well as the importance of preoperative imaging to single-out the precise anatomic mechanisms underlying the clinical manifestations. The other two patients with PCM were successfully treated with BLS, with resolution of symptoms while maintaining excess weight loss. Both BLS and restoration have been widely accepted in the aforementioned consensus process. On the contrary, conversion to SG is not widely accepted and was not performed in our centre. The reason for this is double: (1) SG can also lead to nutritional deficiencies; (2) SG very often leads to suboptimal outcomes and needs further revision. The fact that all cases of PCM were successfully managed underlines the fundamental role of adequate patient follow-up, especially in the first 1–3 years [25–27]. It is the authors’ opinion that this can influence long-term outcomes much more than the surgery itself, and is functional to optimizing each patients’ gains. The team has worked much to avoid losses to follow-up (none in this series) and keep the patients in the stream of active care. As many patients erroneously believe outpatients visits to be a demanding task, we have developed a remote follow-up system that is based essentially in replacing some interval visits with video calls. We believed this has helped maintaining contact with surgical patients and protects them from the bad outcomes of a careless attitude.

In three cases of RWG/IWL, RGP was used, with good results in two and the aforementioned nutritional complication. According to our internal protocol, RGP was performed in cases where the pouch was excessively large, under two specific circumstances: when the biliary limb was already of adequate length (200–250 cm), and/or when the presence of extensive intra-abdominal adhesions precluded the safe mobilization of the intestinal loops. While RGP may make sense when radiologically indicated, there is an extreme paucity of data on this procedure and should be used with caution due to the risk of stenosis [28, 29].

The second most common surgical revision indication was severe medically refractory reflux disease which was seen in 0.6% of OAGB patients. The low incidence testifies to the fact that a properly fashioned pouch (long!) can avoid most reflux-related symptomatology, especially when combined with an anti-reflux mechanism in high-risk cases. However, when this occurs RYGB remains the solution to reflux disease and was successful in 100% of the 20 re-operated patients, in line with other data from the literature (93.8% resolution in 32 patients reported by Kassir et al.) [30]. The proposed algorithm includes the possibility of switching to RYGB (long-limb) also in case of RWG/IWL with coexistent reflux disease.

Finally, rare but insidious complications are anastomotic stenosis and marginal ulcers. Both were treated surgically only after failure of attempted endoscopic management. The preferred approach was anastomosis take-down and re-fashioning, which featured universal resolution of the disease. This last point is of no less interest, especially as it was not considered at all by Kermansaravi et al. [19]. They propose restorative surgery or conversion to RYGB while endoscopic measures are considered inadequate. We suggest that RGJA should be highly considered for the treatment of anastomotic disease in OAGB. In cases of marginal ulcers, the size of the gastric pouch should also be taken into consideration. A large gastric pouch may result in increased gastric acid production [31]; therefore, in such cases, a RGP in addition to the RGJA may be warranted. In the present study, the gastric pouch has never been found to be excessively large, and thus RGP was not required.

Another noteworthy finding emerged when comparing patients undergoing their first revisional bariatric procedure with those who had undergone one or multiple prior bariatric surgeries before the OAGB: the latter were more often re-operated for RWG. These results reinforce the need to regard obesity as a chronic relapsing disease [32]. Patients with a history of previous bariatric or revisional surgery failures are at a significantly higher risk of RWG. Therefore, comprehensive psychologic support aimed at addressing dysfunctional eating behaviors and psychologic patterns is crucial, especially in this high-risk subgroup [33]. Looking ahead, pharmacologic therapy may become an important adjunct to both surgical and psychologic treatment in optimizing long-term outcomes. However, the potential role of pharmacotherapy was beyond the scope of the present study and was not evaluated.

Limitations of the study include its retrospective and monocentric nature which limit generalizability of the results. However, the conduct of the study in a single expert centre could be useful in determining which could be the benchmark outcomes in the field: the best possible results achieved through a standardized patient approach and surgical technique. The sample size may also appear limited but considering the low incidence of surgical revision need, we believe it may be considered entirely adequate. Duration of follow-up is short-term and investigation of long-term follow-up will be not only desirable but also mandatory.

Despite the cited limitations, we believe this study contributes significantly to our knowledge of one anastomosis gastric bypass and the surgical treatment of its long-term complaints, picturing an overall low-morbidity procedure, with a great chance of success of revisional surgery when indicated, featuring few complications, when a rational decisional approach is used. These findings provide valuable insights that can guide bariatric surgeons in selecting the best revisional strategy for patients undergoing OAGB.

## Conclusions

OAGB appears to be a solid procedure with low revision rates. When revision is necessary, high success and low morbidity rates can be accomplished applying a tailored management algorithm. Different revisional procedures should be employed depending on the indication. cRYGB is the best strategy for reflux disease while BLL has optimal results in WR/IWL. Anastomotic stenosis and ulcers may be successfully approached with RGJA. BLS is a good option for severe nutritional deficiencies, while restoration can be taken into consideration when pouch stenosis represents a major factor.

## Data Availability

The data underlying this article cannot be shared publicly to maintain the privacy of individuals who participated in the study. However, data will be shared upon reasonable request to the corresponding author.
